# The life strategy of bacteria rather than fungi shifts in karst tiankeng island-like systems

**DOI:** 10.1128/aem.01581-24

**Published:** 2024-11-26

**Authors:** Cong Jiang, Changchun Qiu, Wei Shui

**Affiliations:** 1 College of Environment and Safety Engineering, Fuzhou University, Fuzhou, China; 2 College of Urban and Environmental Sciences, Peking University, Beijing, China; Colorado School of Mines, Golden, Colorado, USA

**Keywords:** karst tiankeng, fragmented habitat, microbial *r-K *selection theory, abundant taxa, ecological function

## Abstract

Karst tiankeng is a typical terrestrial habitat island-like system, known as an oasis in a degraded karst landscape. However, we know little about the composition, structure, and life strategies of soil microbial communities in the karst tiankeng ecosystem. In this study, we use amplicon sequencing to investigate the soil bacteria and fungi of 26 karst tiankeng in two typical karst tiankeng groups. The results showed that the composition and structure of bacterial and fungal communities were significantly different at two dimensions (among and within the karst tiankeng group). Bacteria showed more sensitivity to variation in the karst tiankeng area and isolation than fungi. With the increase of karst tiankeng area and isolation, the bacterial life strategies shift from *K*-strategist to *r*-strategist, likely due to the changes in soil properties (total phosphorus, Ca, and soil water content). Abundant and rare taxa play different roles in karst tiankeng ecosystems; abundant taxa serve a key role in nutrient cycles and life strategy shifts by occupying the key status in networks. Considering the key role of soil microbes in ecosystems, more attention must be paid to the impact of habitat loss on soil microbial life strategies, particularly in the ecological impact of life strategies change of abundant and rare taxa.

**IMPORTANCE:**

These findings highlight that habitat loss or fragmentation induces a shift in microbial life strategies and improves our understanding of the composition and biogeography of karst ecosystem microorganisms.

## INTRODUCTION

Karst tiankengs are a large-scale surface negative terrain habitat island-like system newly discovered at the beginning of the 21st century and known as “the most spectacular karst landscape on the earth” (1). The karst tiankeng is characterized by its large volume, surrounded by vertical cliffs, and connected to an underground river at the bottom (2). The interior of the karst tiankeng maintains an independent pristine habitat and is a natural complex of geology, climate, soil, animal and plant, and microorganisms (3). Karst tiankengs vary in area and are isolated by vertical cliffs, making them a typical habitat island-like system (4). Our previous studies have confirmed that tiankengs are important “reservoirs for biodiversity conservation” and “species refuges” (5, 6).

The relationship between the species diversity and the island (or island-like) area, known as the species-area relationship (SAR), is among the most general laws in ecology (7, 8). The SAR patterns contribute to understanding how biodiversity is lost as a result of habitat loss (9, 10). However, beyond diversity, microbial functional traits in biogeography remain largely unknown. Soil microbes have great complexity in terrestrial ecosystems, and microbial ecologists have proposed a classification of soil microbial functional traits based on microbial life strategies and growth rates (11, 12). Trait-based microbiota life strategies support crucial soil functions by regulating soil structure and biogeochemical cycle (13, 14). In recent years, a growing body of work involving microbial life strategy analysis has been applied in many environments and provided a key dimension for describing community functions beyond structure and diversity (15–17). For instance, the rocky desertification succession (17), karst vegetation restoration (16), and grassland restoration (18) alter soil microbial life strategies. Microbial trait-based life strategies are key indicators of community functioning and represent interrelated traits due to evolutionary and physiological trade-offs based on environmental conditions (12). To fully understand the soil microbial ecology in karst tiankeng, it is necessary to systematically consider trait-based life strategies. This knowledge can significantly enrich our comprehensive understanding of soil microbial ecology within karst tiankeng ecosystems.

Previous theoretical and empirical studies have demonstrated three main candidate mechanisms that contribute to positive SAR: sampling effect, area *per se* effect, and habitat heterogeneity effect (19). Generally, the *K*-strategists are dominate disturbed in oligotrophic environments and are characterized by slow growth rates (11), while *r*-strategists are mainly disturbed in labile nutrient fractions and are characterized by rapid growth rates (20). Following this logic, the larger island harboring high-quality habitats than smaller island would facilitate the shift from *K*- to *r*- strategists. Smaller islands are usually characterized by stronger edge effects, which harbor lower soil moisture or nutrient habitats (21). In addition, previous studies indicated that island remoteness favors AM fungal communities’ life history characteristics (22). However, there is a significant difference in the isolation characteristics of karst tiankengs from ocean islands. Thus, whether habitat loss can lead to a shift in microbial life strategies is an important and unanswered question.

Bacterial and fungal communities are the main components of the soil microbiota and exhibit distinctly different life strategies and morphological characteristics (23), leading to disparate sensitivities to spatial changes. Previous studies indicated that soil bacteria and fungi exhibit similar biogeographic patterns but different mechanisms (21). In addition, soil microbiota are composed of a small number of abundant taxa and a large number of rare taxa (24). Heterogeneity in substrate preference and adaptation to environmental stresses is an important reason for the differences in soil microbial abundance (25). Abundant taxa occupy a wide niche width and are more resilient to environmental challenges. Previous studies primarily aimed at the entire microbial community life strategies (26, 27) and ignored the differences in life strategies of abundant and rare taxa.

In this study, we investigated the spatial scaling of bacterial and fungal composition, structure, and functional traits, using microbial data (bacteria and fungi) collected from 26 karst tiankengs in two typical karst tiankeng groups in China (Dashiwei and Zhanyi). We aimed to answer three key questions: (i) Whether abundant and rare taxa have different response patterns to the unique habitats of karst tiankengs? (2) How do the life strategies of soil microbes shift with karst tiankeng area and isolation? (3) What are the key factors influencing the life strategies of soil microbes?

## MATERIALS AND METHODS

### Study sites and sampling

This study was conducted at the two typical karst tiankeng groups, including dashiwei tiankeng group (DSW) in Guangxi Province (24°30′–25°03′N, 106°10′–106°51′E) and zhanyi tiankeng group (ZY) in Yunnan Province (25°35′–25°57′N, 103°29′–103°39′E). These two karst tiankeng groups are distributed in the biodiversity hotspot areas of China and have preserved a systematic and complete tiankeng evolution chain. The dense distribution and variety of tiankengs are ideal areas for island biogeographic research. The climate type of DSW is mid-subtropical monsoon, with an average annual temperature of 16.6°C and average annual precipitation of 1140 mm. The main vegetation types were evergreen broad-leaved forest and evergreen deciduous broad-leaved mixed forest. The soil type is mainly neutral or alkaline limestone soils. The climate type of ZY is subtropical plateau monsoon, with an average annual temperature of 14.5°C and average annual precipitation of 1008 mm. The main vegetation types were evergreen broad-leaved forests and coniferous forests. The soil type is mainly red soil.

A total of 12 (DSW) and 14 (ZY) karst tiankengs were selected as our study sites (Fig. S1). We calculated the area of karst tiankeng using ArcGIS 10.7 (ESRI, Inc., Redlands, CA, USA). Isolation was measured as the karst tiankeng depth and measured using real-time kinematic. The morphological data of the 26 karst tiankengs are presented in Table S1. Sampling was carried out in September 2022. On each karst tiankeng, we established two to six 10 ×  10  m^2^ plots. Three 1 ×  1  m^2^ quadrats were randomly set in each plot, and soil samples were collected from the 0–15-cm depth using the five-point sampling method. The three quadrat soil samples were mixed to form one composite soil sample, and a total of 93 soil samples were obtained. The fresh soil samples were transported in ice boxes and stored in the laboratory for further analysis. The soil samples were divided into three parts, one part was placed at −80°C for DNA extraction, one part was used for soil moisture content determination, and the remaining portions of soil were naturally air-dried for soil physicochemical property analysis.

### High-throughput sequencing

Soil microbial DNA was extracted using the CTAB methods. The extracted DNA concentration and purity were determined using the 1% agarose gel electrophoresis. For bacteria, PCR amplification of the V4-V5 region was performed using primer pair 515F (5′-GTGCCAGCMGCCGCGGTAA-3′)/907R(5′-CCGTCAATTCCTTTGAGTTT-3′). For fungi, PCR amplification of the ITS1-1F region was performed using primer pair ITS1-1F-F (5′-CTTGGTCATTTAGAGGAAGTAA-3′)/ITS1-1F-R (5′-GCTGCGTTCTTCATCGATGC-3′) (28, 29). Using the Bio-Rad T100 gradient PCR instrument, the PCR reaction was performed with Phusion Master Mix (2×), forward primers (0.2 µM/µL), reverse primers (0.2 µM/µL), gDNA (1 ng/µL), and sterile water. The PCR amplification procedure was as follows: pre-denaturing at 98°C for 1 min; the 30 cycles included 98°C, 10 sec; 50°C, 30 sec; 72°C, 30 sec; and 72°C, 5 min. The PCR product was detected by agarose gel electrophoresis at a concentration of 2%, and after mixing the samples at an equal concentration according to its concentration, the PCR product was purified by agarose gel electrophoresis at a concentration of 1 × TAE and 2% and cut glue recovery target strips. PCR product purification was performed using the Qiagen gel recovery kit (Qiagen, Germany). Libraries were constructed using the TruSeq DNA PCR-free sample preparation kit (Illumina, USA), qualifying the libraries were quantitated and tested by Qubit 4.0 fluorometer (Invitrogen, Thermo Fisher Scientific, OR, USA).

The qualified libraries were sequenced on the NovaSeq 6000 PE250 (Illumina, San Diego, CA, USA). The raw data were quality-controlled and merged using the QIIME2 programs (v2021.2). The DADA2 plug-in in the Qiime2 software was applied to the filter, denoising, merge processes, and clustered into amplicon sequence variant (ASV) (30). The QIIME2 feature-classifier was used for taxonomic annotations of the bacterial and fungal species and aligned to the GREENGENES2 (v2022.10) and UNIT (v8.2) databases by references (31, 32).

### Soil properties

Soil water content was determined by soil sample weight stabilization after drying at 105°C. Soil pH was measured in a suspension of soil and deionized water at a ratio of 1:2.5. Soil organic matter content was assessed using the modified Walkley–Black procedure involving potassium dichromate oxidation. Dissolved organic carbon in the soil was analyzed by adding distilled water (at a ratio of 5:1) to 3 g of soil, followed by centrifugation after agitation (250 r/min, for 1 hour), and detection using a TOC analyzer. The total nitrogen content in the soil was determined using the semimicro Kjeldahl method (Kjeltec 2200 Auto Distillation Unit, FOSS, Hillerød, Sweden). Forest hydrolytic nitrogen (LY/T1229-1999) assay was employed to measure available nitrogen in the soil. Total and available phosphorus levels were measured via colorimetric analysis using an ultraviolet-visible spectrophotometer (UV-2550, Shimadzu, Kyoto, Japan). Calcium and magnesium concentrations were extracted from Mehlich-III solution and quantified using inductively coupled plasma emission spectrometry (Optima 2100 DV, Perkin-Elmer, Waltham, MA, USA).

### Statistical analyses

Abundant and rare taxa of soil microbes were defined following the previous study (33). Briefly, ASVs with average relative abundance of >0.05% across all samples were regarded as “abundant”; ASVs with average relative abundance of <0.01% across all samples were regarded as “rare.”

The *rrn* operon copy number estimation is employed using the *rrnDB* database to determine whether the bacterial community adopts *K*-strategies or *r*-strategies. A lower and higher *rrn* operon copy number indicates *K*-strategies and *r*-strategies, respectively (34). Fungal life strategies were classified by phylum level and ecological guild level. FUNGuild database was utilized to estimate the ecological guild of each fungal ASVs (35), with *Ectomycorrhizal* and *Saprophytic* fungi classified as *K*- and *r*-strategists, respectively (36). In addition, oligotrophic bacterial taxa (*K*-strategists) include *Acidobacteria*, *Actinobacteria*, *Planctomycetes*, and *Chloroflexi*. Eutrophic bacterial taxa (*r*-strategists) include *Bacteroidetes*, *Gemmatimonadetes*, and *Firmicutes* (37). *Basidiomycota* and *Ascomycota* were designated as oligotrophic fungi taxa (*K*-strategists) and eutrophic fungi taxa (*r*-strategists), respectively (38).

The bacterial and fungal communities alpha-diversity analysis was conducted in R. Principal coordinate analysis (PCoA) was used to visualize bacterial and fungal communities within the DSW and ZY karst tiankeng groups. When constructing networks, we set the strong Spearman’s correlation (|r| > 0.7 for bacteria, |r| > 0.5 for fungi) (39), and visualization was conducted using Gephi software (0.10.1; Gephi, WebAtlas, France). Linear regression was employed to evaluate the relationship between area and isolation on the microbial life strategies on each karst tiankeng. To improve model fit, the data were log-transformed. The structural equation modeling (SEM) was used to elucidate the causal pathways through which karst tiankeng island biogeographic factors and soil physicochemical properties influence the life strategies of soil microbes. Structural equation modeling was conducted via the lavaan package in R.

## RESULTS

### Composition of soil bacterial and fungal communities in the karst tiankeng

Regarding the bacterial communities, a total of 7,634,262 high-quality reads were clustered into 643,509 ASVs. There were 219 and 30,132 ASVs belonging to abundant and rare taxa, respectively. For the fungal communities, a total of 6,444,618 high-quality reads were clustered into 68,200 ASVs. Among these fungal ASVs, 341 and 13,011 ASVs were defined as abundant and rare taxa, respectively. The PCoA and Veen results revealed that bacterial and fungal community composition (total, abundant, and rare) in karst tiankeng exhibited differences between individual karst tiankeng and between two karst tiankeng groups (Fig. S2 to S4). The shared bacterial ASVs of the total, abundant, and rare taxa in the DSW and ZY occupy a higher proportion, while the unique fungal ASVs of the total and rare taxa to each tiankeng were higher than the shared ASVs.

**Fig 1:**
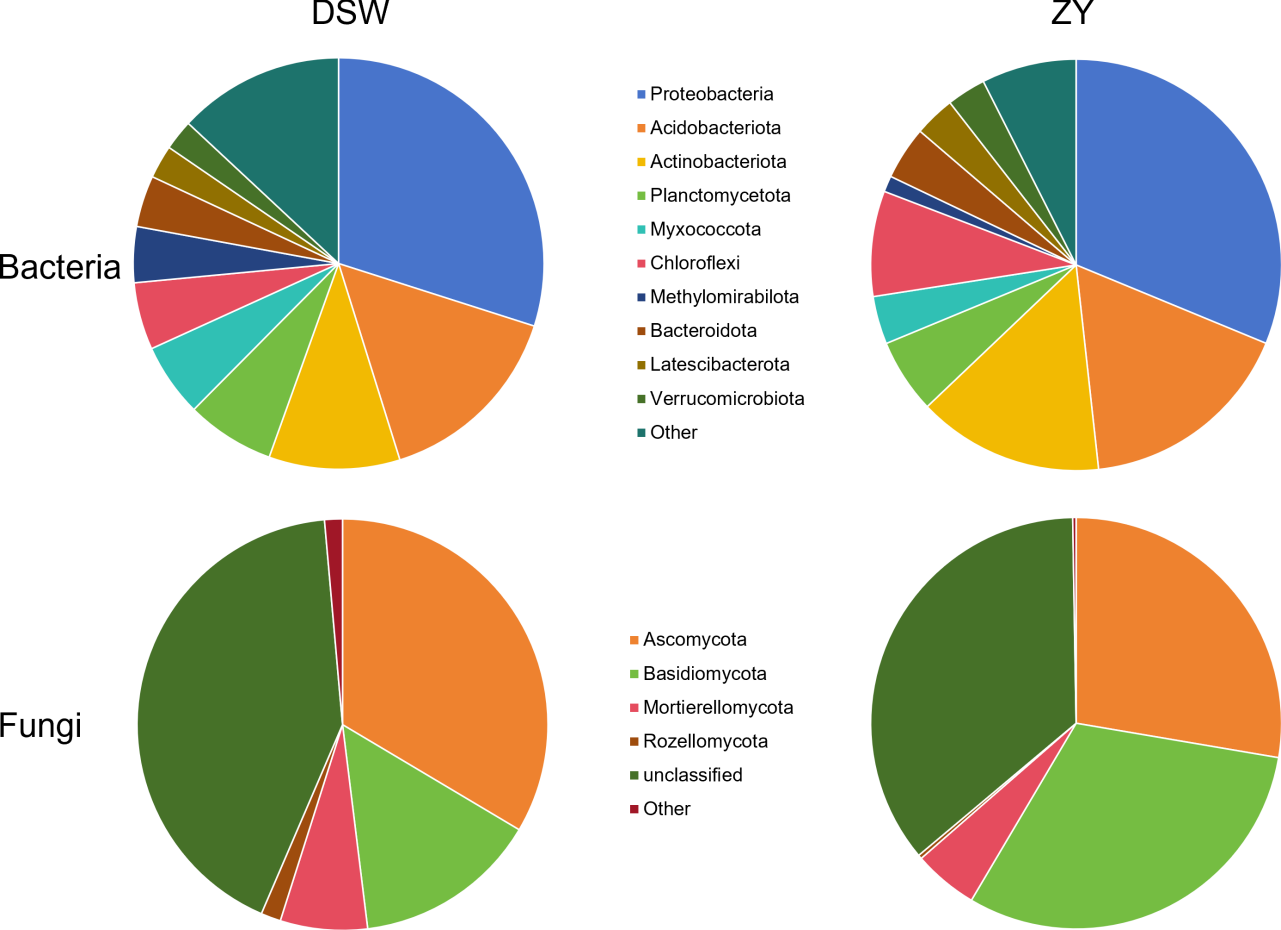
Composition of bacterial and fungal communities at the phylum level in the DSW and ZY karst tiankeng group.

At the phylum level, *Proteobacteria*, *Acidobacteriota*, and *Actinobacteriota* were the most important components of the soil bacterial communities (Fig. 1). *Ascomycota*, *Basidiomycota*, and *Mortierellomycota* were the most abundant fungal taxa across all soil samples.

### The effect of karst tiankeng area and isolation on the soil microbe life strategy

**Fig 2:**
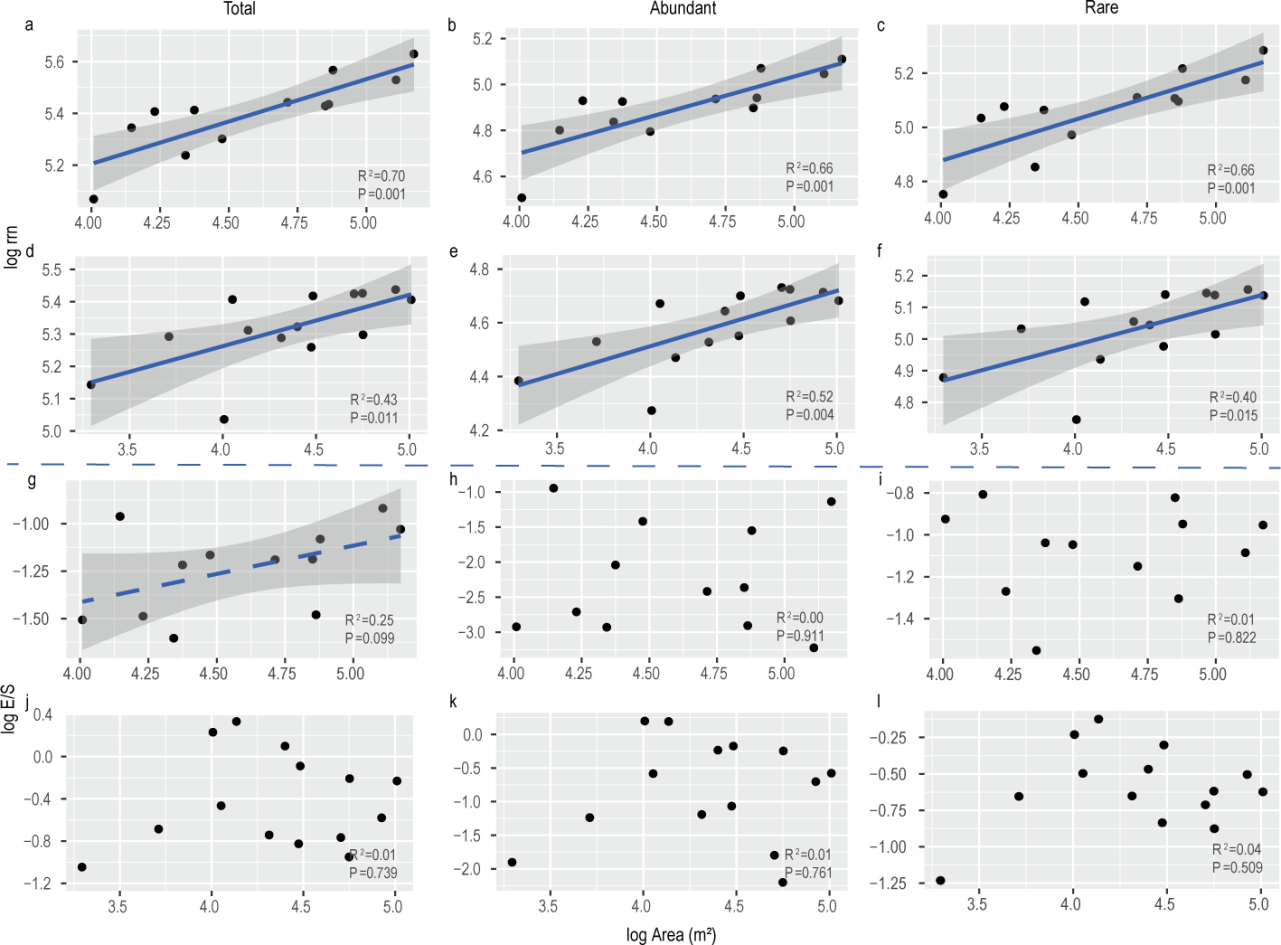
Relationships between karst tiankeng area and 16S rRNA (*rrn*) operon copy number (**a–f**) or ectomycorrhizal and saprotrophic fungi ratio (E/S) (**g–l**). Panels a–c and g–i are for DSW and d–f and j–l are for ZY. Bacterial life strategies are indicated by the 16S rRNA (*rrn*) operon copy number, and fungal life strategies are indicated by the ectomycorrhizal and saprotrophic fungi ratio. The solid lines represent significant linear regressions (*P*  <  0.05), and the dashed lines represent marginally significant regressions (*P*  <  0.10).

The linear regression exhibited that total, abundant, and rare taxa of bacterial *rrn* operon copy number increased significantly with karst tiankeng area (Fig. 2). The increase in *rrn* operon copy number indicated a shift in the bacterial community from *K*- to *r*-strategists. Soil fungi, however, showed markedly different life strategist patterns. Karst tiankeng area did not affect fungal life strategy. The above relationships were robust for different taxa (total, abundant, and rare taxa) estimators.

The bacterial *rrn* operon copy number exhibited positive relationships with karst tiankeng isolation (Fig. S5). The increase of karst tiankeng depth promoted the shift of the bacterial community from *K*- to *r*-strategists. Karst tiankeng isolation did not affect fungal life strategists.

### Coexistence patterns of bacterial and fungal communities in the karst tiankeng

**Fig 3:**
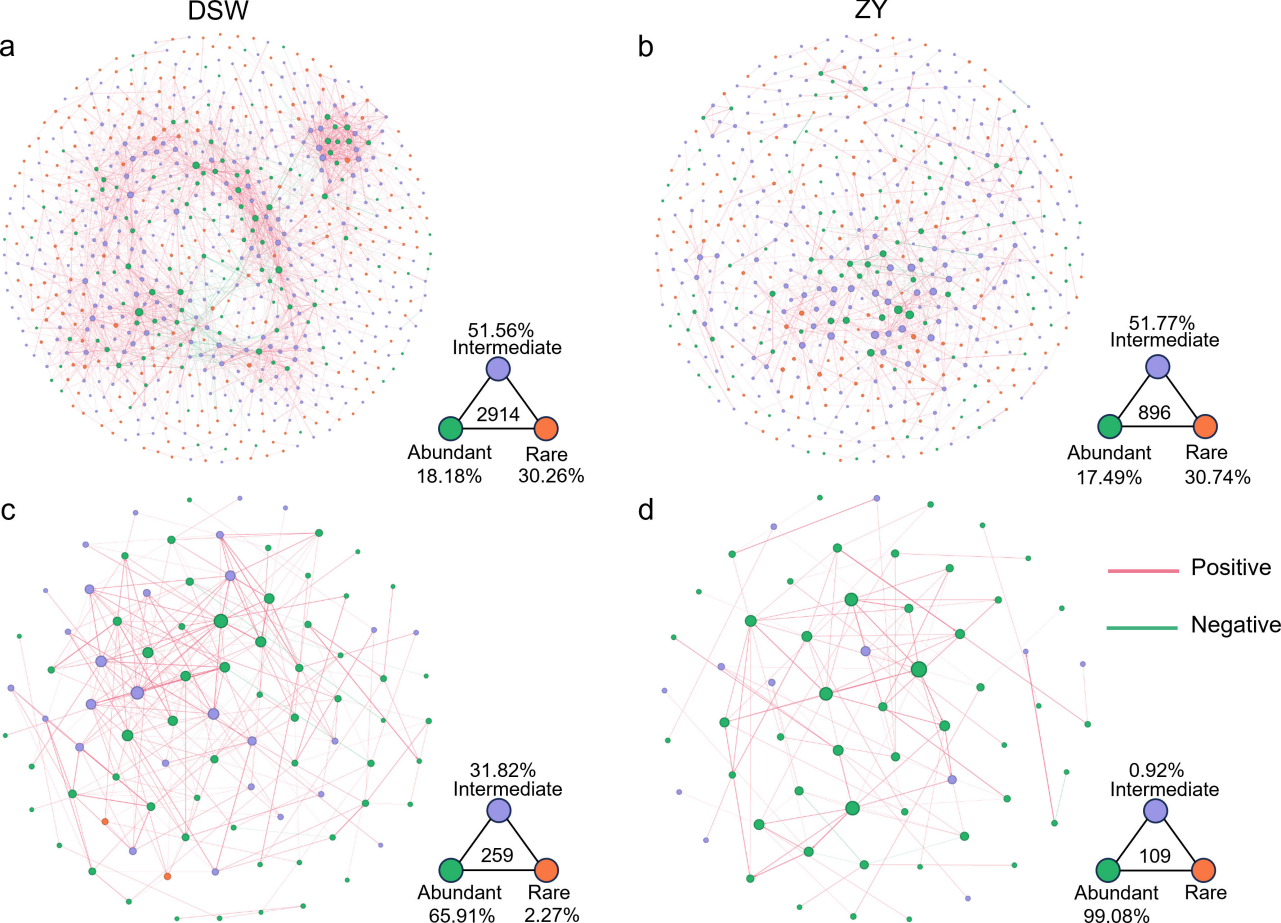
Co-occurrence patterns of the soil bacterial (**a, b**) and fungal (**c, d**) communities in DSW and ZY karst tiankeng group.

Co-occurrence networks of bacterial communities in the karst tiankeng soils consisted of 737 nodes and 2914 edges in DSW, which is higher than in ZY (nodes = 566, edges = 896, Fig. 3a and b). Regarding DSW and ZY bacterial networks, the proportions of rare nodes (30.26% and 30.74%) were higher than abundant nodes (17.49% and 18.18%). Co-occurrence networks of fungal communities consisted of 88 nodes and 259 edges in DSW and 60 nodes and 109 edges in ZY (Fig. 3c and d). Conversely, the proportions of fungal abundant nodes were higher than rare nodes.

To determine the topological roles of karst tiankeng soil microbial, the *Zi-Pi* plot result is shown in Fig. S6. Twelve and six taxa were detected as “keystones” in bacterial and fungal networks, respectively. These bacterial taxa belong to *Proteobacteria*, *Acidobacteriota* (*K*-strategists), *Actinobacteriota* (*K*-strategists), and *Gemmatimonadota* (*r*-strategists), and fungal taxa belong to *Ascomycota* (*r*-strategists) and *Mortierellomycota* (*r*-strategists). All keystone taxa belong to abundant taxa among the bacterial and fungal networks (Table S2).

### Linking bacterial and fungal communities to potential functions

**Fig 4:**
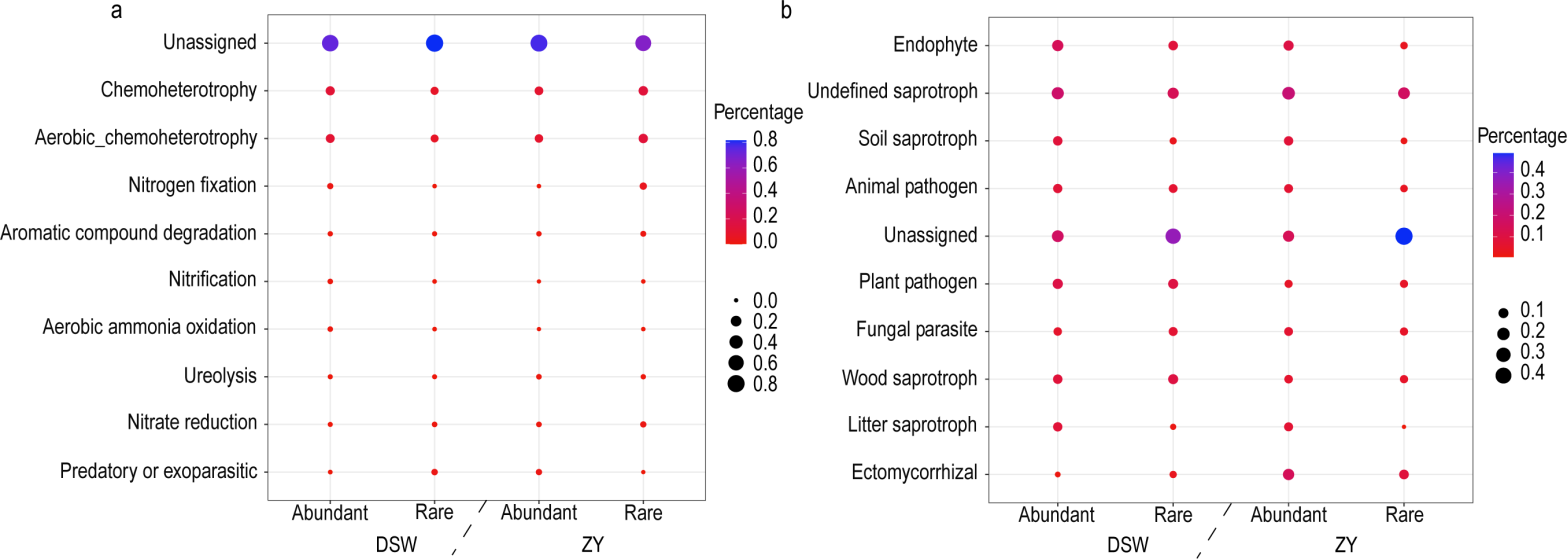
The potential functions of the abundant and rare microbial taxa. (**a**) Bacteria and (**b**) fungi. The color represents the potential function abundance of the sample (abundant or rare taxa), and the bubble size represents the percentage of a specific potential function in the sample.

The potential functions of the abundant and rare bacteria showed that the abundant taxa involved in higher activity at major metabolic functions (Fig. 4), such as chemoheterotrophy, aerobic chemoheterotrophy, nitrogen fixation, aerobic ammonia oxidation, and nitrification. In comparison, rare taxa contain more diversity of potential functions (Fig. S7). The fungal potential function results showed that fungal abundant taxa were mainly involved in endophyte, plant pathogen, and soil saprotroph. The fungal rare taxa contain more diversity of potential functions, and a large proportion (>30%) were unassigned.

**Fig 5:**
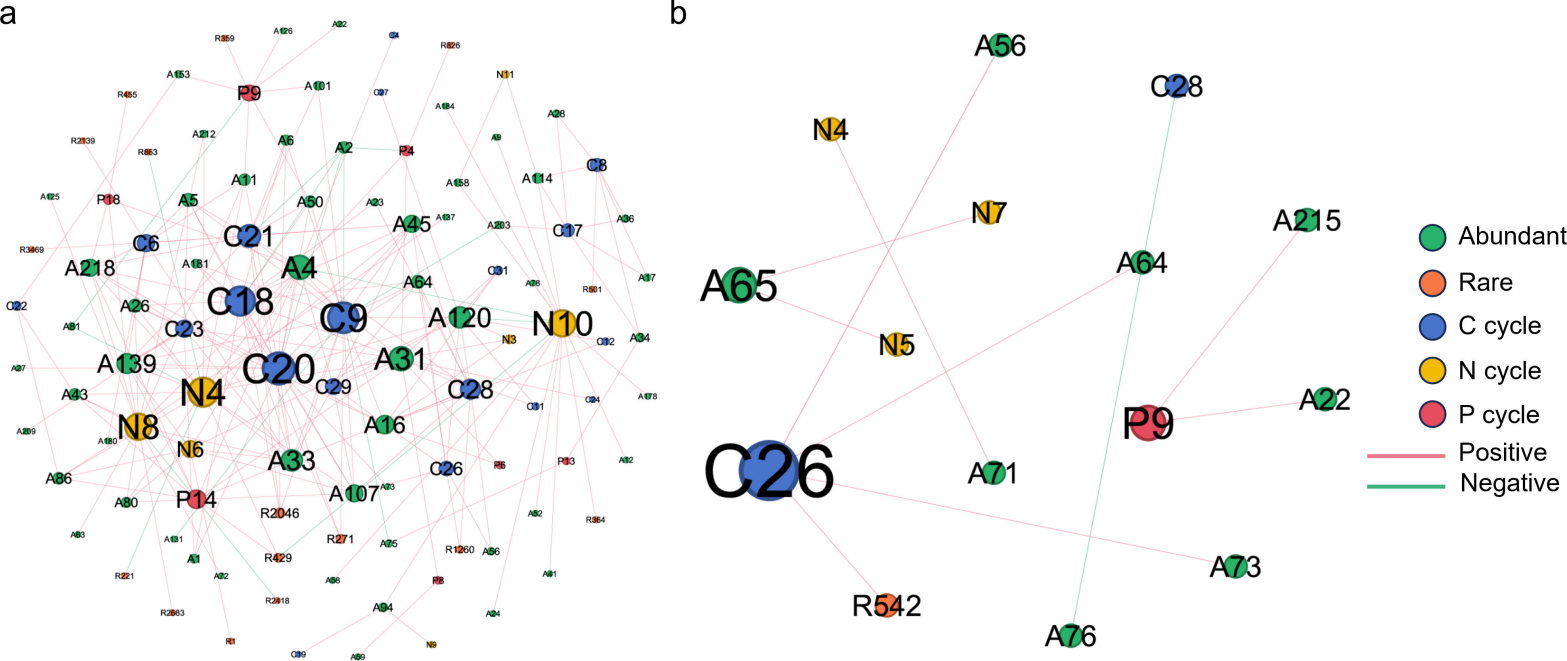
Co-occurrence patterns of functional genes of the C, N, and P cycles and their potential hosts in the dashiwei (**a**) and zhanyi (**b**) karst tiankeng group soils.

Furthermore, we explored the relationship between the C, N, and P cycles and bacterial abundance and rare taxa (Fig. 5). Network analysis exhibited that abundant bacteria were highly associated with C, N, and P cycles. The 33 functional genes in DSW were highly associated with 57 bacterial abundant taxa, while only 6 functional genes in ZY were highly associated with 8 bacterial abundant taxa. The nodes shared by the two networks belong to the phyla of *Firmicutes*, *Actinobacteriota*, and *Acidobacteriota*. The functional genes shared by the two networks belong to the C cycle (fermentation to acetate and fermentation to ethanol), N cycle (assimilatory nitrate reduction), and P cycle (cytochrome aa3-600 menaquinol oxidase) (Table S3).

### Responses of soil microbial community composition, function, and life strategies to environmental variables

**Fig 6:**
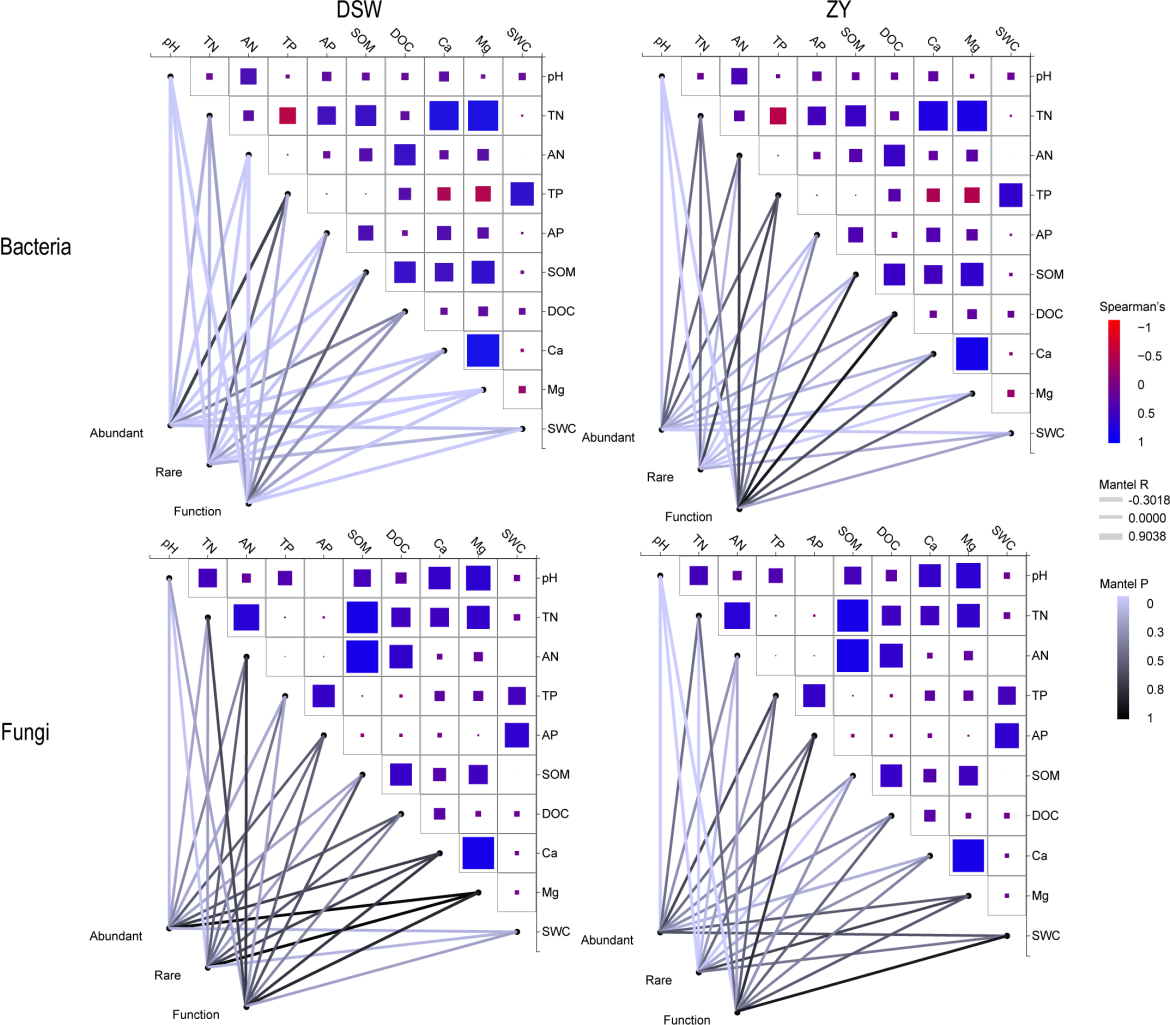
Mantel tests of each environmental variable on the abundant and rare taxa and functional genes in the DSW and ZY karst tiankeng group soils. TN, total nitrogen; AN, available nitrogen; TP, total phosphorus; AP, available phosphorus; SOM, soil organic matter; DOC, dissolved organic carbon; SWC, soil water content.

Our study found differences in soil physicochemical properties between different karst tiankeng (Table S4). The total phosphorus and soil water content of DSW and the dissolved organic carbon and soil water content of ZY increased significantly with the karst tiankeng area (Table S5). Furthermore, we tested the effect of environmental factors on the soil microbial community. The Mantel test results exhibited responses to environmental variables of taxa and function in DSW were more significant than those in ZY (Fig. 6). The response of abundant and rare taxa to environmental variables was inconsistent for both bacterial and fungal communities. For both bacterial and fungal communities, pH was notably associated with abundant, rare, and functional genes. Four environmental variables (AP, SOM, Ca, and Mg) were also associated with rare taxa of bacterial and fungal communities. More environmental variables were strongly related to the rare taxa than the abundant taxa. Abundant bacterial life strategies were significantly associated with SWC in DSW and ZY (Table S6), while rare bacterial life strategies and abundant and rare fungal life strategies exhibited no obvious association with SWC.

**Fig 7:**
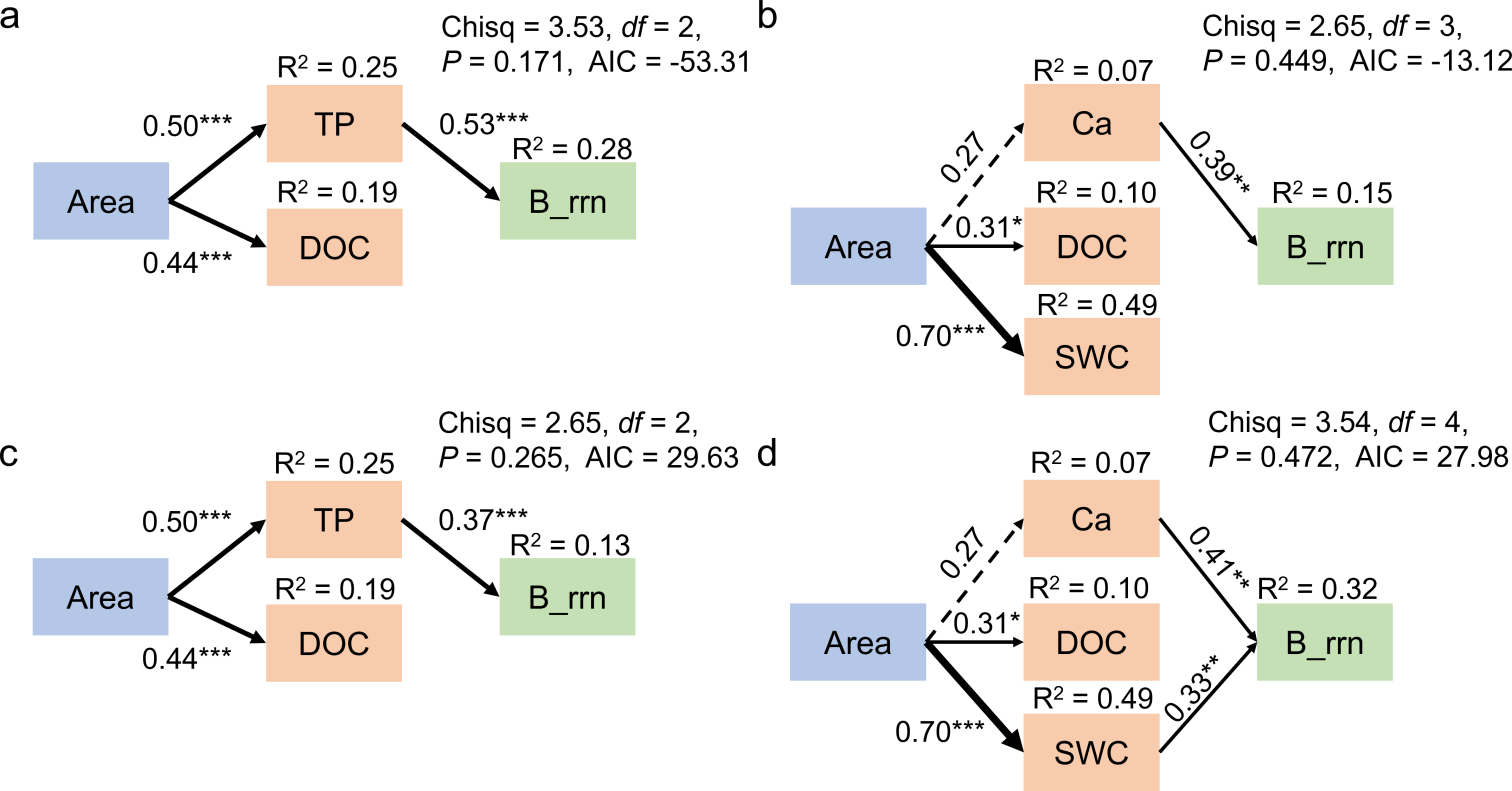
Based on the structural equation model, the direct and indirect effects of karst tiankeng area on life strategies of total (a, b) and abundant (c, d) taxa of soil bacterial pathways were analyzed. Panels a and c are for DSW, and panels b and d are for ZY. The black solid arrows in the model indicate significant positive pathways (^*^P < 0.05, ^**^P < 0.01, ^***^P < 0.001, respectively). The black dashed arrows represent marginally significant positive pathways (*P*  <  0.10). The line thickness represents the standardized path coefficients. *R^2^* represents the proportion of variance explained by the dependent variable. TP, total phosphorus; DOC, dissolved organic carbon; SWC, soil water content.

The SEM was applied to reveal the direct and indirect pathways that influence the life strategies of soil bacterial communities (Fig. 7). The SEM results revealed that the area did not have a direct effect on bacterial life strategies. In DSW, the great *rrn* operon copy number of the total and abundant bacterial taxa on larger karst tiankeng was driven by higher TP on those karst tiankeng (Fig. 7a and c). In ZY, the great *rrn* operon copy number of total bacterial taxa on larger karst tiankeng was driven by higher Ca content (Fig. 7b), while the great *rrn* operon copy number of abundant bacterial taxa on larger karst tiankeng was mainly driven by the increased Ca and SWC; DOC did not affect the bacterial life strategies (Fig. 7d).

## DISCUSSION

### Community compositions and structure of abundant and rare taxa in the karst tiankeng

In our study, the bacterial communities in the karst tiankeng were dominated by *Proteobacteria and Acidobacteriota* (Fig. 1). *Proteobacteria* participate in ecological and phylogenetic values and play a crucial role in energy metabolism (40). *Acidobacteriota* species are considered to perform the function of organic matter decomposition and nutrient cycles (41). In addition, the fungal abundant phyla, including *Ascomycota* and *Basidiomycota*, may play a key role in soil aggregation and nutrient uptake (42). This result suggested that microbes involved in the nutrient cycle and energy metabolism might survive well in karst tiankeng. However, no significant differences were observed between the two karst tiankeng groups. This phenomenon may illustrate that these soil microbes were well adapted to the habitat conditions of karst tiankeng.

The bacteria and fungal community structures exhibited differences between individual karst tiankeng and between two karst tiankeng groups (Fig. S2). Regarding the bacterial and fungal abundant taxa, there was no significant change among the different individual karst tiankeng, suggesting that bacterial and fungal abundant taxa were ubiquitous across all karst tiankeng soils (Fig. 3 and 4). Abundant microbes occupy wide niches and exhibit the characteristics of ecological persistence and effectively adapt to altered environments (33). However, the bacterial and fungal rare taxa were exhibit not evenly distributed in karst tiankeng soils. These endemic rare microbes were mainly due to the unique habitat of each karst tiankeng.

Complex co-occurrence networks reveal microbial interactions with specific ecological niches (43). In both bacterial and fungal co-occurrence networks (Fig. 3), the proportion of positive associations was dominant, indicating the occurrence of microbial mutualism to adapt to the habitat conditions of karst tiankeng. The abundant taxa occupy the central position of co-occurrence networks, which facilitates the growth of other species. The keystone species in microbial co-occurrence networks mainly belong to *Proteobacteria*, *Actinobacteriota*, and *Gemmatimonadota* (bacteria) and *Ascomycota* and *Mortierellomycota* (fungi) (Table S2). Diverse species occupy different ecological niches and are involved in different ecological processes (38, 39). These findings indicate that abundant and rare taxa have distinct distributions, and keystone species may play an important role in regulating community function.

### Bacterial rather than fungal life strategies are associated with karst tiankeng area and isolation

In this study, we found that the soil bacterial *rrn* operon copy number exhibited positive relationships with karst tiankeng area. Specifically, in the small karst tiankeng, the bacterial *rrn* operon copy number is low (Fig. 2). The increase in *rrn* operon copy number indicated that the karst tiankeng area increased shifted the bacterial community from *K*- to *r*-strategists and indicated that the bacterial community favored the *r*-strategy in bigger karst tiankeng. Furthermore, our study is consistent with previous studies exhibiting that microbial life strategies are closely related to soil properties (17, 18). Small karst tiankengs are characterized by lower vegetation coverage and intense edge effects, resulting in a decrease in soil water content and nutrients and appearing to be more conducive to bacterial communities with relatively slow renewal rates (*K*-strategists). Mantel tests and Spearman correlation analysis also confirmed these results (Fig. 6). The relatively wetter and rich labile nutrient environments of large karst tiankeng favor *r*-strategists with relatively fast renewal rates (Table S6). In the microbial network, keystones belong to abundant taxa, such as *Acidobacteriota* (*K*-strategists), *Gemmatimonadota* (*r*-strategists), *Ascomycota* (*r*-strategists), and *Mortierellomycota* (*r*-strategists), and play a key role in the shift in microbial life strategy. Our findings suggest that soil property is an important determinant of bacterial life strategies in karst tiankeng.

The soil bacterial *rrn* operon copy number also exhibited positive relationships with karst tiankeng depth (isolation). The more isolated karst tiankeng should maintain the internal microclimate, and the warming and humidification effect is more significant (4). Previous studies have shown that *K*-strategists are more inclined to live in arid soil ecosystems and tend to develop various functions to adapt to arid conditions (15). In our study, the more isolated karst tiankengs are characterized by higher soil moisture. In addition, previous studies have confirmed that human interference can lead to shifts in soil microbial life strategies (44). Due to the isolation of vertical cliffs, the more isolated karst tiankeng, the more conducive to reducing human interference. Unlike bacteria, the fungal life strategies were not affected by the karst tiankeng area and isolation. The previous studies also indicated that soil properties influenced bacterial trait-based strategies, but did not limit fungal life strategies (45). Together, our results suggest that the area and isolation of karst tiankeng affect the life strategies of bacteria, not fungi.

### Functional attributes of abundant and rare taxa in the karst tiankeng

Microbial communities are core contributors to ecosystem function and play an important role in biogeochemical processes, including nutrient cycling and fixation (46). By annotating the functions of abundant and rare taxa, the abundant taxa showed higher activity in some major metabolic functions, while the rare taxa contained more diversity of metabolic functions (Fig. 4). Furthermore, the potential hosts for distinct functional genes were identified by network analysis, and the results exhibited that the most abundant taxa were the key potential hosts of functional genes for the biogeochemical cycle (e.g., C, N, and P cycles) in karst tiankengs (Fig. 5). Our study reveals that abundant bacteria dominate the biogeochemical functions in karst tiankeng soils. Similarly, previous studies have found that abundant bacteria usually act as active contributors in biogeochemical cycles (39, 47). The bacterial *rrn* operon copy number is often regarded as an indicator of nutrient utilization efficiency and survival strategies for individual organisms (26, 48). The rare taxa had a higher *rrn* operon copy number than the abundant taxa, suggesting that abundant taxa had a stronger nutrient utilization efficiency. Our network analysis results also confirmed these results (Fig. 3). Abundant taxa included fewer species, and their biochemical functions were diverse. In contrast, rare taxa possess relatively homogeneous biogeochemical functions with a higher number of species. These results indicate that different life strategies of abundant and rare taxa result in differences in metabolic capacity and growth rate (39).

Through network analysis, our result showed that *K*-strategists represented by *Actinobacteriota* and *Acidobacteriota* and *r* strategists represented by *Firmicutes* were widely involved in soil C, N, and P cycling in karst tiankengs. Habitat loss is accompanied by a shift in the life strategies of soil bacteria. The abundant bacteria in small karst tiankeng behaved more like *K*-strategists and preferred affinity for recalcitrant C substrates. The abundant bacteria in small karst tiankeng are more easily adapted to environmental fluctuations (48). Small karst tiankeng is characterized as more susceptible to disturbance from the outside of the karst tiankeng environment (e.g., rocky desertification) and human interference (49). Habitat loss leads to the reduction of habitat quality and promotes the shift of soil bacteria from *r*-strategist to *K*-strategist. *K*-strategists can achieve maximum growth efficiency in resource-constrained environments, but this higher metabolic capacity may lead to an increased rate of nutrient depletion, leading to ecosystem degradation (50, 51).

### Conclusions

In this study, we conducted an amplicon sequencing to investigate the composition, structure, and life strategies of soil bacteria and fungi in karst tiankeng. *Proteobacteria* and *Acidobacteriota* (bacteria) and *Ascomycota* and *Basidiomycota* (fungi) were the most abundant phyla across all samples. The bacterial and fungal abundant taxa were ubiquitous in all karst tiankengs, while rare taxa exhibited a high proportion of unique species per individual karst tiankeng because of the heterogeneous habitat. The increase of karst tiankeng area and isolation shifted the bacterial community towards *r*-strategy, and among-karst tiankeng differences in soil properties generate the bacterial life strategies-area relationship. However, fungal life strategies did not exhibit a significant correlation with karst tiankeng and isolation. Abundant taxa play a keystone role in the co-occurrence network and are closely related to nutrient cycling and shift of bacterial life strategy. It is worth noting that some unmeasured factors may influence microbial life strategies, including historical factors and vegetation characteristics. Further studies need to systematically consider the multiple factors that influence microbial life strategies. Overall, our study has implications for a better understanding of the characteristics of soil bacterial and fungal communities and the shift of life strategies in the karst tiankeng ecosystem.

## Data Availability

The DNA sequences were uploaded to the NCBI SRA under the accession numbers PRJNA1132395 (bacteria) and PRJNA1132403 (fungi).

## SUPPLEMENTAL MATERIAL

Supplemental material aem.01581-24-s0001.docx Figures S1 to S7; Tables S1 to S6.

ASM does not own the copyrights to Supplemental Material that may be linked to, or accessed through, an article. The authors have granted ASM a non-exclusive, world-wide license to publish the Supplemental Material files. Please contact the corresponding author directly for reuse.
