# Supplementary material for: The life strategy of bacteria rather than fungi shifts in karst tiankeng island-like systems
Source: Appl Environ Microbiol. 2024 Nov 26;90(12):e01581-24. doi: 10.1128/aem.01581-24 (PMC11653732; doi:10.1128/aem.01581-24)
Supplement: Supplemental material — Figures S1 to S7; Tables S1 to S6. [file aem.01581-24-s0001.docx]

**Supplement files**


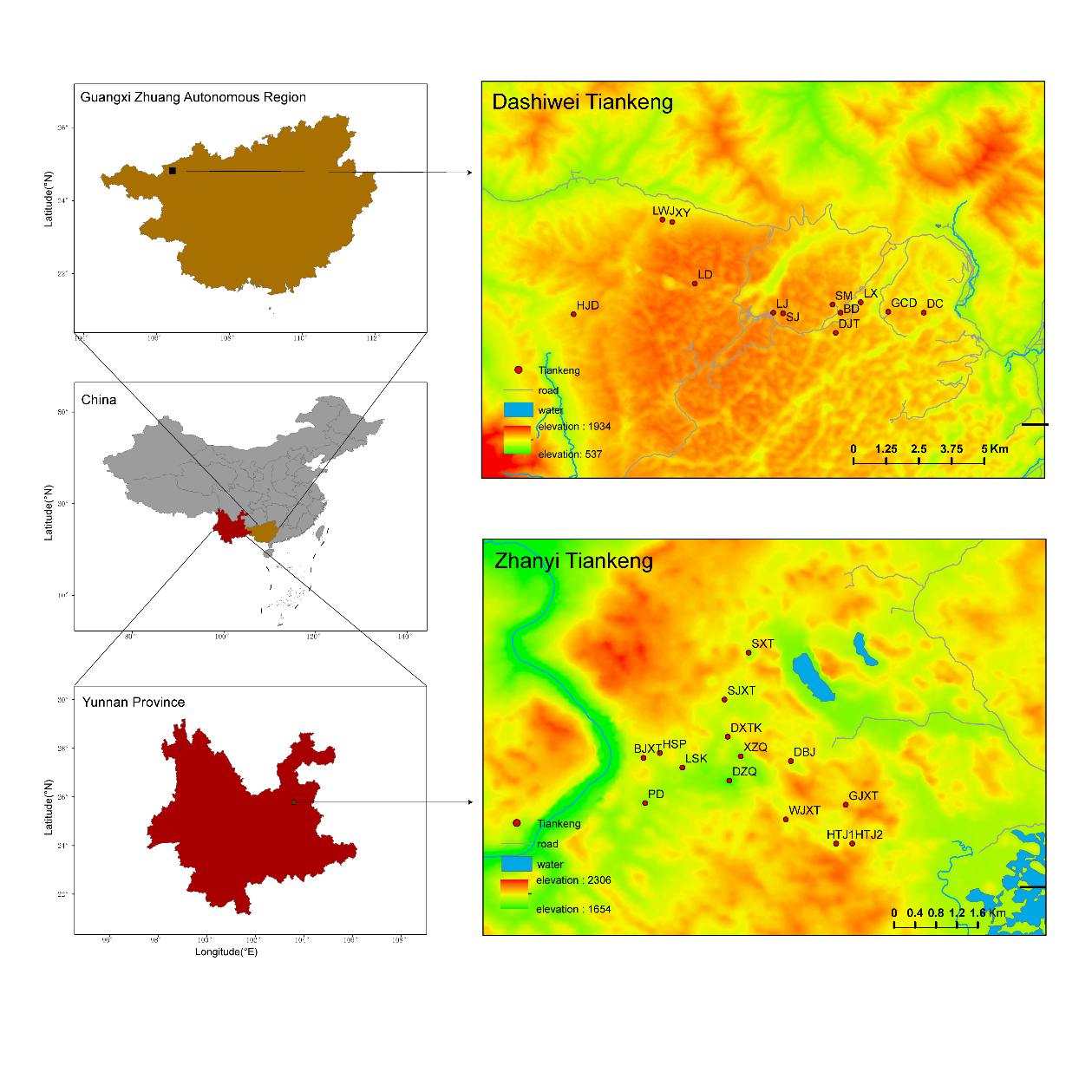


Fig. S1. Location of study site on the map of China. Dashiwei tiankeng group: LJ: Luojia, XY: Xuanya, LD: Ladong, BD: Baidong, SJ: Shujia, DC: Dacao, HJD: Huangjingdong, SM: Shenmu, GCD: Chuandong, LWJ: Laowuji, DJT: Dengjiatuo, LX: Liuxing; Zhanyi tiankeng group: LSK: Laoshenkeng, WJXT: Wangjiaxiantang, XZQ: Xiaozhujing, DXTK: Daxiantangkou, PD: Piandong, HSP: Huoshipo, HTJ2: Huataojing2, GJXT: Gaojiaxiantang, BJXT: Bajiaxiantang, HTJ1: Huataojing1, DZJ: Dazhujing, SJXT: Shaojiaxiantang, DBJ: Dabaiji, SXT: Shenxiantang karst tiankeng.


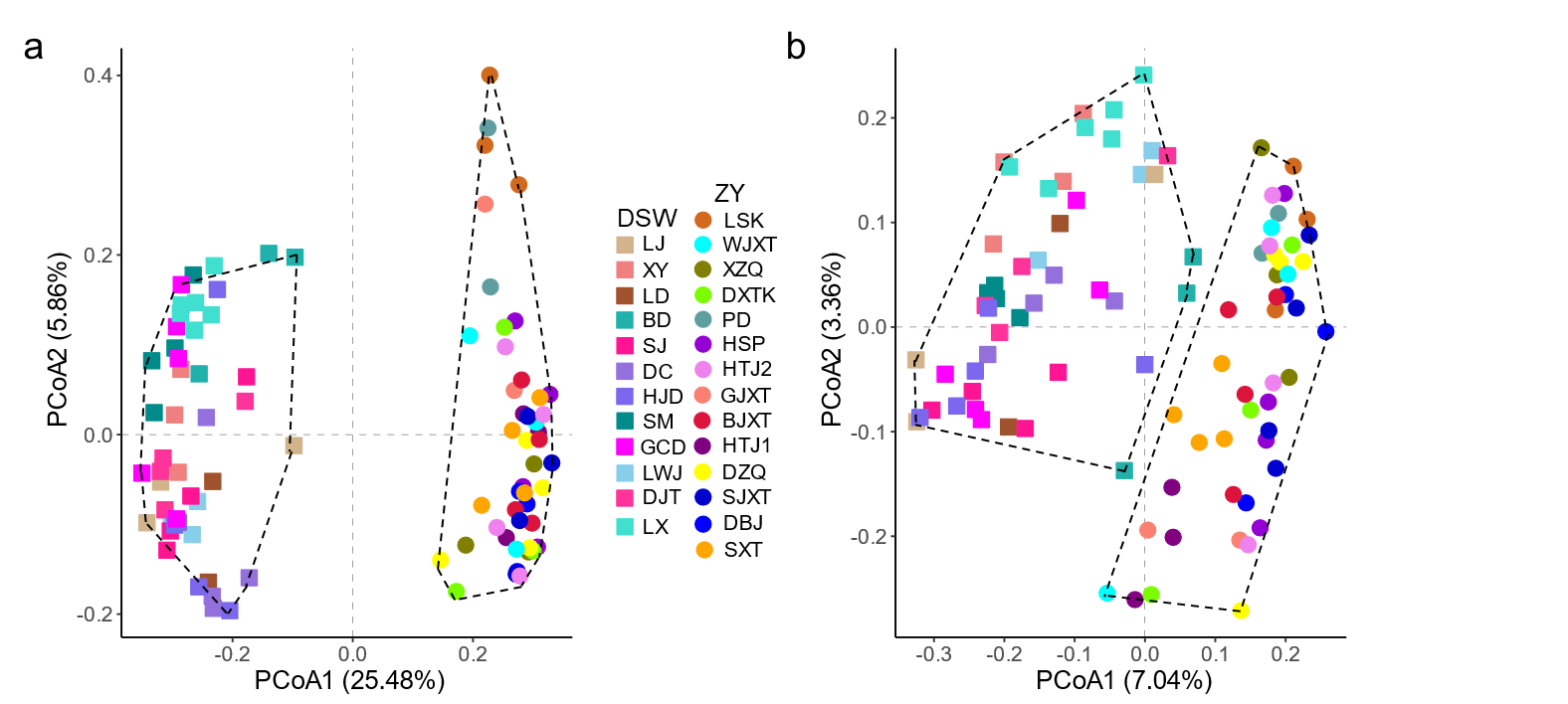


Fig. S2. Bacterial (a) and fungal (b) community compositions in the karst tiankeng revealed by PCoa.


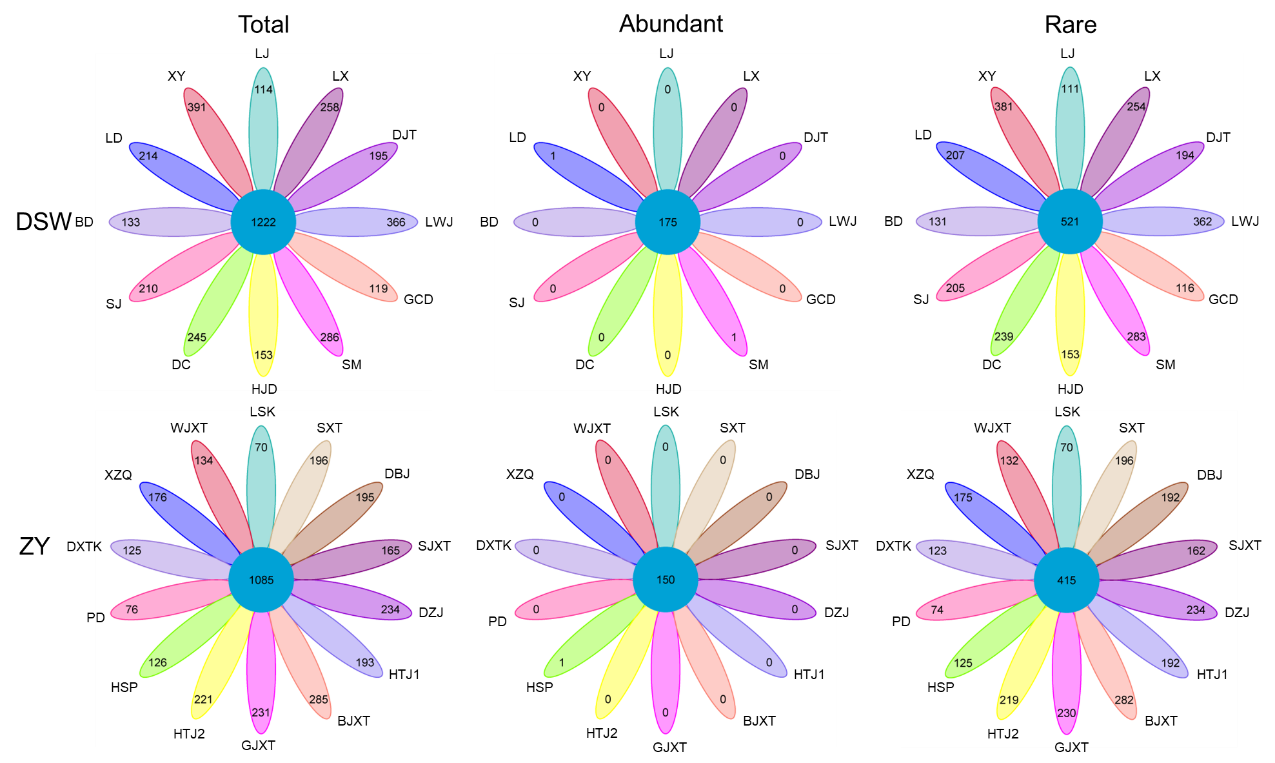


Fig. S3. Bacterial composition of the total, abundant and rare taxa in the dashiwei (DSW) and zhanyi (ZY) karst tiankeng groups.


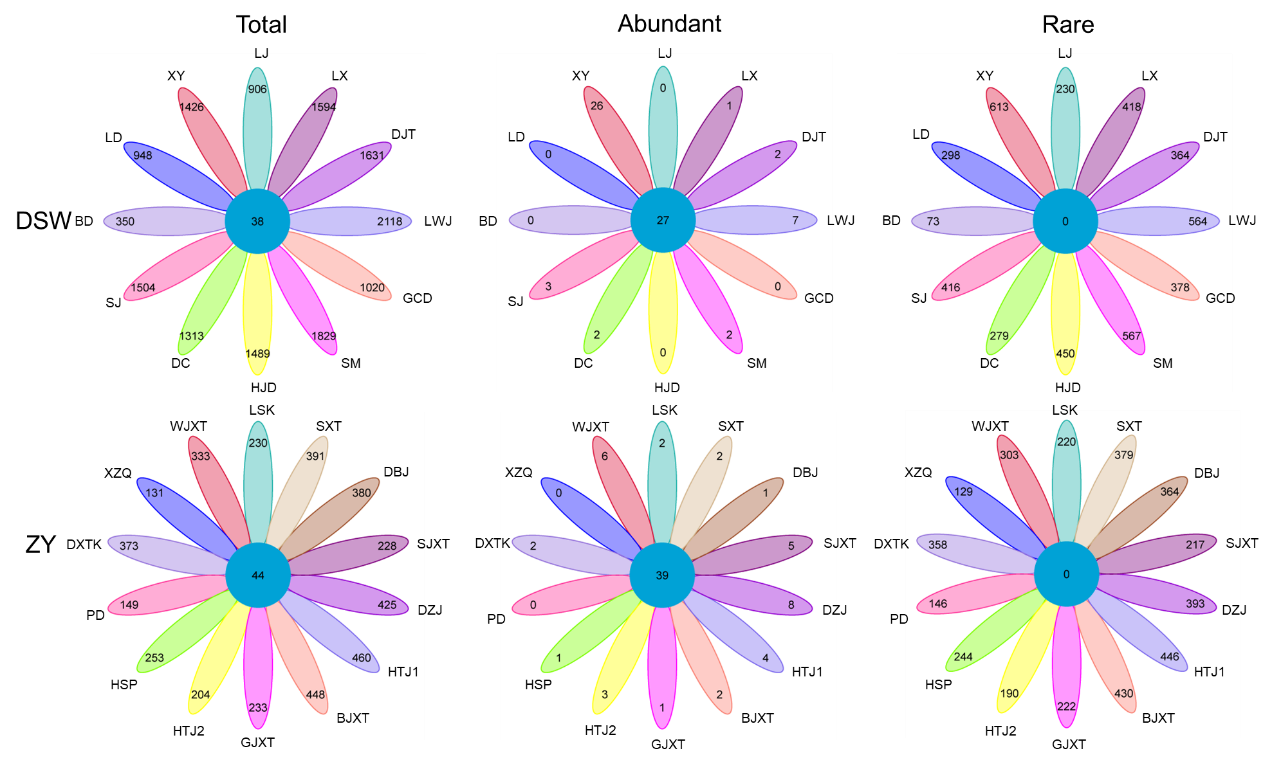


Fig. S4. Fungal composition of the total, abundant and rare taxa in the dashiwei (DSW) and zhanyi (ZY) karst tiankeng groups.


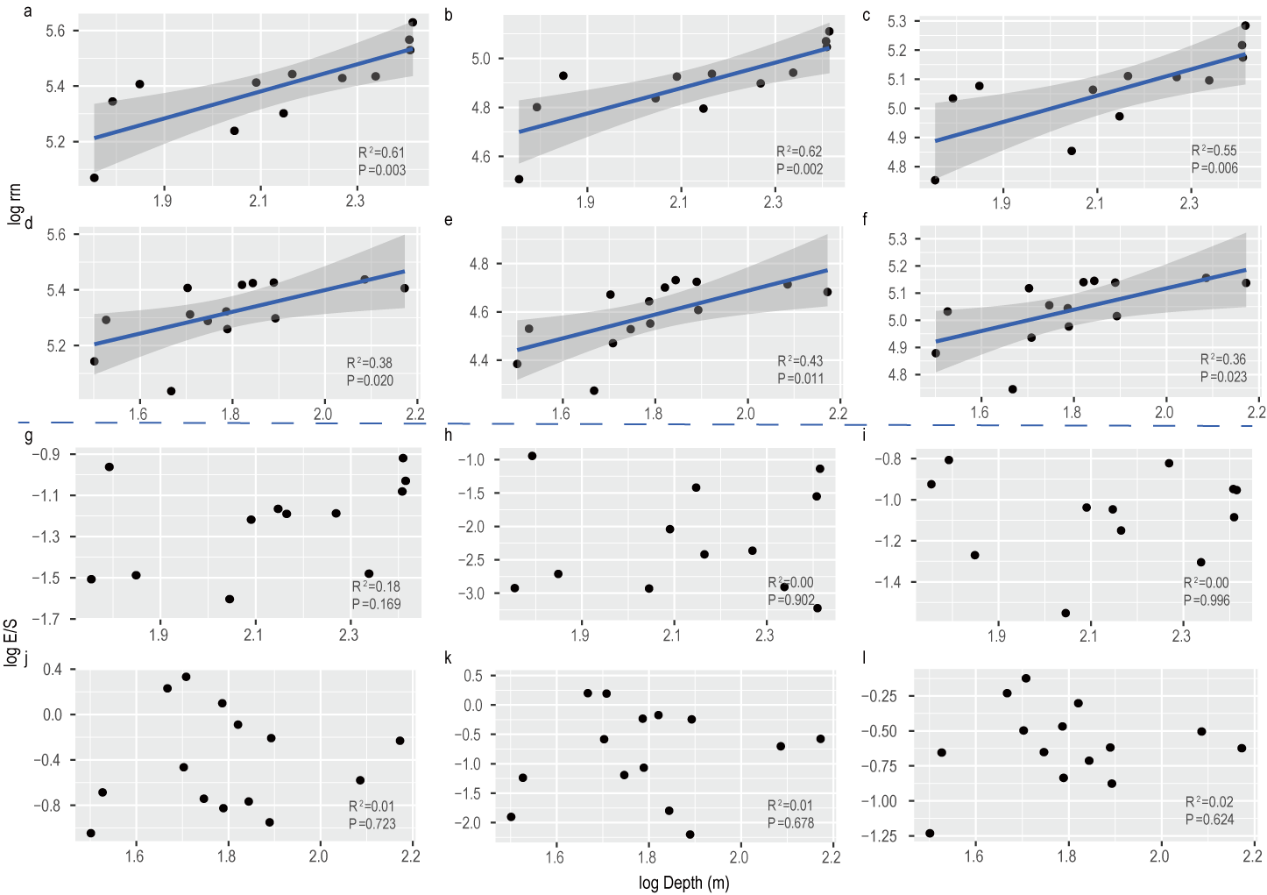


Fig. S5 Relationships between karst tiankeng isolation (depth) and 16S rRNA (*rrn*) operon copy number (a-f) or ectomycorrhizal and saprotrophic fungi ratio (E/S) (g-l).. Panels a-c and g-i are for dashiwei (DSW), d-f and j-l are for zhanyi (ZY). Bacterial life strategies are indicated by the 16S rRNA (*rrn*) operon copy number, and fungal life strategies are indicated by the ectomycorrhizal and saprotrophic fungi ratio. The solid lines represent significant linear regressions (*P* < 0.05), and the dashed lines represent marginally significant regressions (*P* < 0.10).


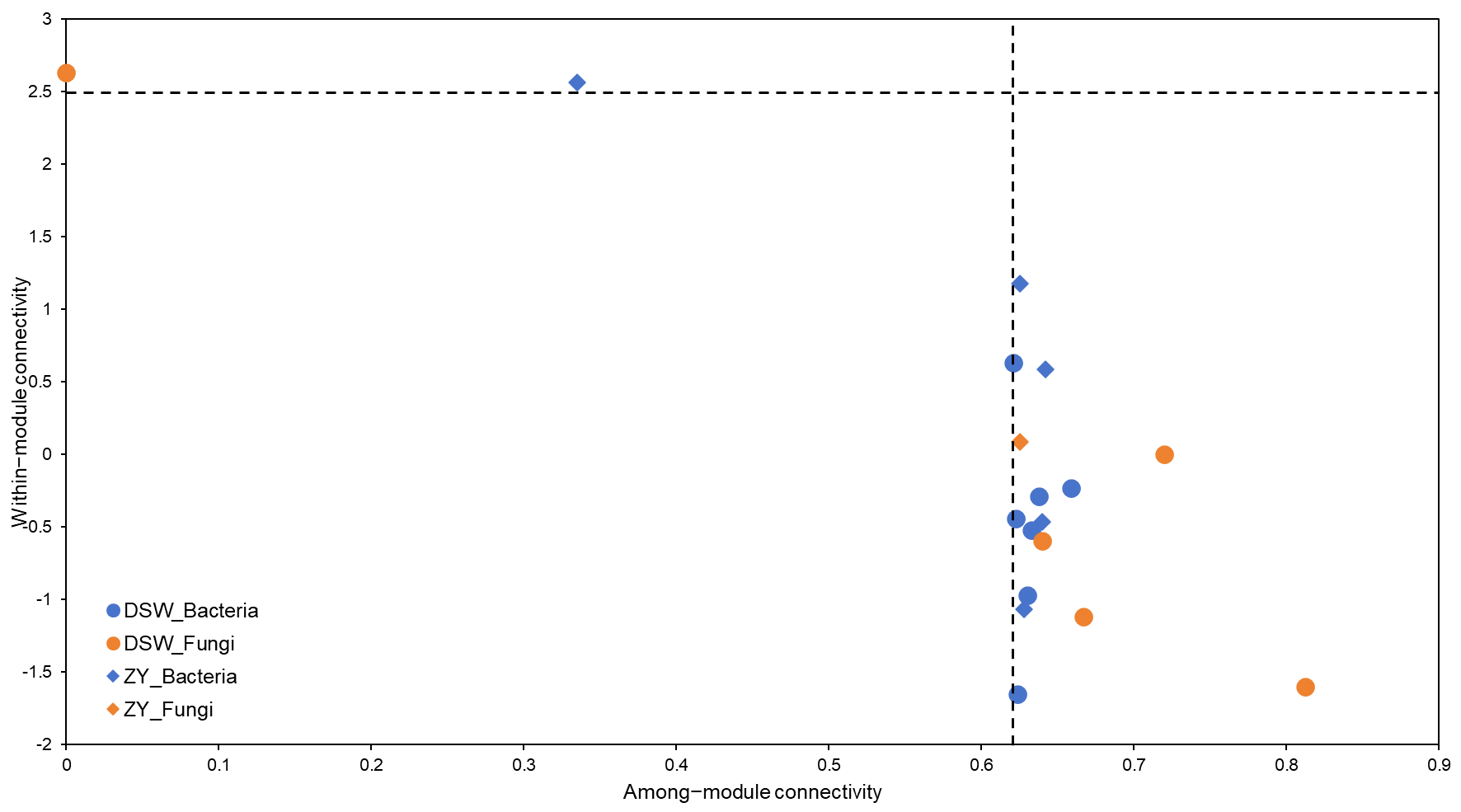


Fig. S6. The distribution of bacterial and fungal taxa based on their topological roles. Each symbol represents a taxa in bacterial and fungal networks.


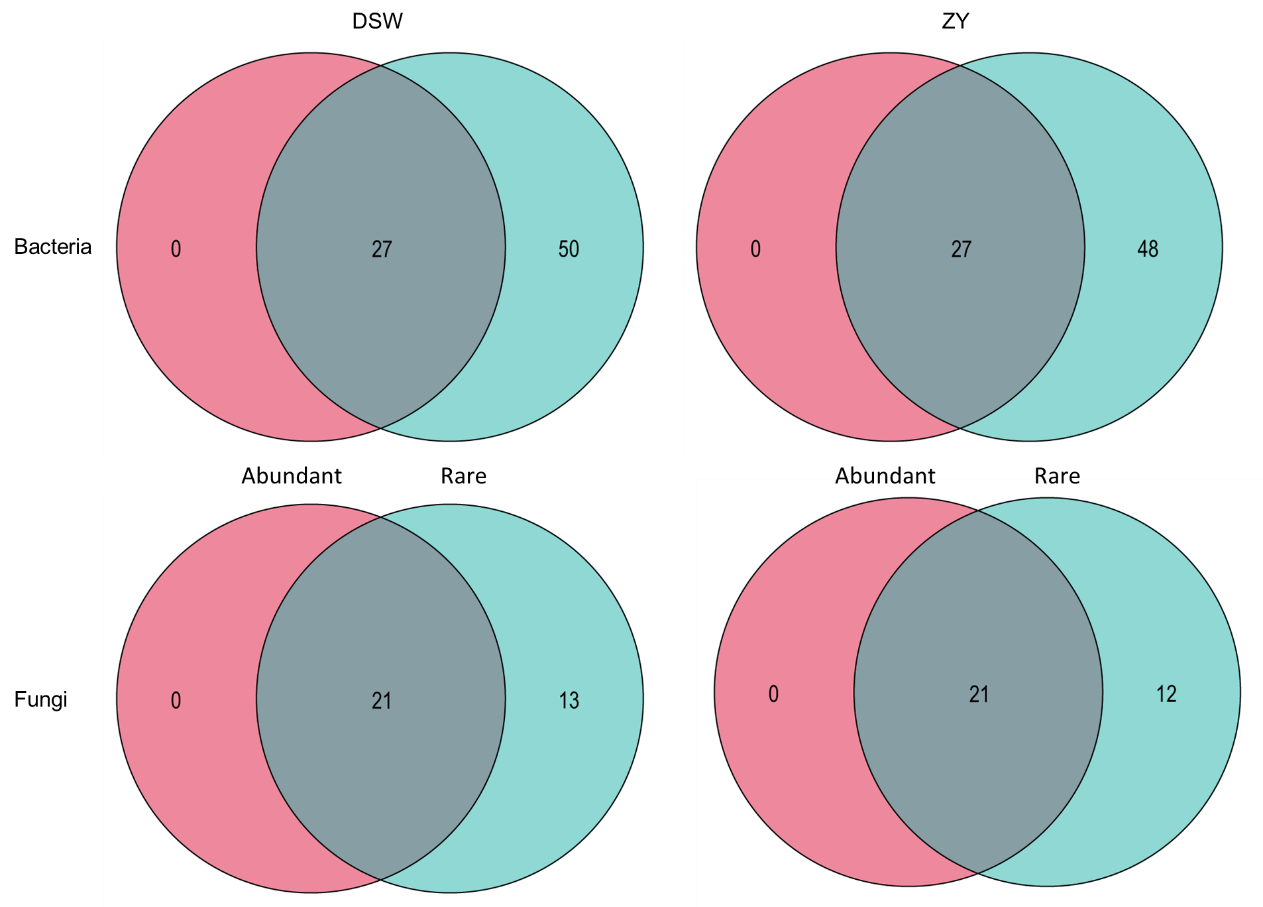


Fig. S7. The potential functions of bacterial and fungal of abundant and rare taxa in the dashiwei (DSW) and zhanyi (ZY) karst tiankeng groups.

Table S1 The area and isolation data of the 26 karst tiankengs.

| Tiankeng | | Area  (m^2^) | Isolation  (m) |
| --- | --- | --- | --- |
| DSW | LJ | 10200 | 70.59 |
|  | XY | 14000 | 62.00 |
|  | LD | 17000 | 146.24 |
|  | BD | 22000 | 260.08 |
|  | SJ | 23700 | 110.97 |
|  | DC | 29900 | 56.82 |
|  | HJD | 51700 | 140.31 |
|  | SM | 70900 | 185.75 |
|  | GCD | 73000 | 255.82 |
|  | LWJ | 75600 | 123.15 |
|  | DJT | 128200 | 217.81 |
|  | LX | 148800 | 256.80 |
| ZY | LSK | 1969 | 38.96 |
|  | WJXT | 5146 | 77.50 |
|  | XZQ | 10167 | 55.79 |
|  | DXTK | 11273 | 28.63 |
|  | PD | 13665 | 33.62 |
|  | HSP | 20597 | 46.50 |
|  | HTJ2 | 25135 | 31.71 |
|  | GJXT | 29821 | 50.44 |
|  | BJXT | 30434 | 69.80 |
|  | HTJ1 | 50608 | 51.06 |
|  | DZJ | 56125 | 78.11 |
|  | SJXT | 56496 | 121.90 |
|  | BJT | 84458 | 61.16 |
|  | SXT | 102388 | 148.70 |

DSW: Dashiwei tiankeng group, ZY: Zhanyi tiankeng group.

Table S2. Information of keystones in bacterial and fungal networks.

|  |  | name | Taxa | Degree | BC | CC | EC | Abundance | Zi | Pi | Phylum | Taxa_roles |
| --- | --- | --- | --- | --- | --- | --- | --- | --- | --- | --- | --- | --- |
| GX | Bacteria | ASV11 | Abundant | 30 | 20 | 0.01 | 0.72 | 0.36 | -0.29 | 0.64 | Proteobacteria | Connectors |
|  |  | ASV20 | Abundant | 18 | 50 | 0.01 | 0.18 | 0.29 | -1.65 | 0.62 | Proteobacteria | Connectors |
|  |  | ASV43 | Abundant | 42 | 61 | 0.01 | 0.86 | 0.24 | -0.23 | 0.66 | Proteobacteria | Connectors |
|  |  | ASV80 | Abundant | 44 | 125 | 0.01 | 0.88 | 0.16 | 0.63 | 0.62 | Proteobacteria | Connectors |
|  |  | ASV108 | Abundant | 29 | 24 | 0.01 | 0.56 | 0.13 | -0.52 | 0.63 | Proteobacteria | Connectors |
|  |  | ASV130 | Abundant | 27 | 44 | 0.01 | 0.42 | 0.13 | -0.44 | 0.62 | Proteobacteria | Connectors |
|  |  | ASV166 | Abundant | 26 | 42 | 0.01 | 0.50 | 0.11 | -0.97 | 0.63 | Acidobacteriota | Connectors |
|  | Fungi | ASV3 | Abundant | 8 | 1231 | 0.00 | 0.14 | 0.13 | -1.60 | 0.81 | Ascomycota | Connectors |
|  |  | ASV15 | Abundant | 10 | 410 | 0.00 | 0.04 | 0.53 | 2.63 | 0.00 | Ascomycota | Module hubs |
|  |  | ASV37 | Abundant | 5 | 214 | 0.00 | 0.05 | 0.66 | -0.59 | 0.64 | Ascomycota | Connectors |
|  |  | ASV61 | Abundant | 5 | 408 | 0.00 | 0.01 | 0.49 | 0.00 | 0.72 | Ascomycota | Connectors |
|  |  | ASV203 | Abundant | 3 | 133 | 0.00 | 0.01 | 0.19 | -1.12 | 0.67 | Ascomycota | Connectors |
| YN | Bacteria | ASV15 | Abundant | 11 | 20 | 0.01 | 0.15 | 0.21 | -1.07 | 0.63 | Proteobacteria | Connectors |
|  |  | ASV66 | Abundant | 20 | 43 | 0.01 | 0.18 | 0.22 | 2.56 | 0.34 | Actinobacteriota | Module hubs |
|  |  | ASV87 | Abundant | 15 | 59 | 0.01 | 0.22 | 0.17 | -0.46 | 0.64 | Proteobacteria | Connectors |
|  |  | ASV133 | Abundant | 20 | 70 | 0.01 | 0.38 | 0.14 | 1.18 | 0.63 | Gemmatimonadota | Connectors |
|  |  | ASV168 | Abundant | 9 | 64 | 0.01 | 0.11 | 0.12 | 0.59 | 0.64 | Proteobacteria | Connectors |
|  | Fungi | ASV8 | Abundant | 8 | 552 | 0.00 | 0.06 | 0.68 | 0.09 | 0.63 | Mortierellomycota | Connectors |

BC: Betweenness centrality, CC: Closeness centrality, EC: Eigenvector centrality.

Table S3. Information of common keystones in co-occurrence network of functional genes and their potential hosts.

|  | ID | Node properties |
| --- | --- | --- |
| Abundant taxa | A22 | *p_Firmicutes; c_Bacilli; o_Bacillales; f_Bacillaceae; g_Bacillus* |
|  | A56 | *p_Actinobacteriota; c_Actinobacteria; o_Propionibacteriales; f_Nocardioidaceae; g_Nocardioides* |
|  | A64 | *p_Acidobacteriota; c_Vicinamibacteria; o_Vicinamibacterales* |
|  | A73 | *p_Actinobacteriota; c_Thermoleophilia; o_Gaiellales; f_Gaiellaceae; g_Gaiella* |
| C cycle | C26 | Fermentation to acetate, lactate -> acetate |
|  | C28 | Fermentation to ethanol, acetyl-CoA to acetylaldehyde (reversible) |
| N cycle | N4 | Assimilatory nitrate reduction, nitrite -> ammonia (NIT-6 or nirA) |
| P cycle | P9 | Cytochrome aa3-600 menaquinol oxidase |

Table S4 Soil properties of the dashiwei (DSW) and zhanyi (ZY) karst tiankeng groups.

| Group | pH | TN (g/kg) | AN (mg/kg) | TP (mg/kg) | AP (mg/kg) | SOM (g/kg) | DOC (mg/kg) | Ca (g/kg) | Mg (g/kg) | SWC (%) |
| --- | --- | --- | --- | --- | --- | --- | --- | --- | --- | --- |
| DSW | 6.74±0.63a | 3.46±1.34b | 282.59±75.21b | 615.66±125.40a | 11.00±5.61b | 70.76±30.70a | 337.41±86.53a | 2.13±1.60b | 0.19±0.16b | 31.02±6.20a |
| ZY | 7.11±0.59a | 6.37±3.33a | 362.15±87.25a | 575.21±125.82a | 78.62±50.00a | 84.98±32.28a | 365.38±192.2a | 7.58±8.20a | 0.56±0.50a | 33.33±5.24a |

TN: total nitrogen, AN: available nitrogen, TP: total phosphorus, AP: available phosphorus, SOM: soil organic matter, DOC: dissolved organic carbon, SWC: soil water content.

Table S5 The influences of karst tiankeng area on soil properties.

| Soil properties | DSW | | | ZY | |
| --- | --- | --- | --- | --- | --- |
|  | *R^2^* | *P* | *R^2^* | | *P* |
| TP | 0.39 | 0.029 |  | |  |
| DOC |  |  | 0.52 | | 0.004 |
| SWC | 0.60 | 0.003 | 0.72 | | 0.001 |

Table S6. Spearman correlation analysis of SWC with abundant (A) and rare (R) bacteria (B) and fungal (F) life-history strateigies in the dashiwei (DSW) and zhanyi (ZY) karst tiankeng groups (*indicates *P*<0.05, **indicates *P*<0.05).

|  |  |  | SWC |
| --- | --- | --- | --- |
|  | DSW | A_B_rrn | **0.433**** |
|  |  | A_F_E.S | -0.104 |
|  |  | R_B_rrn | 0.203 |
|  |  | R_F_ES | -0.150 |
|  | ZY | A_B_rrn | **0.298*** |
|  |  | A_F_E.S | -0.157 |
|  |  | R_B_rrn | 0.080 |
|  |  | R_F_ES | -0.089 |
